# Compartment-specific m^7^G RNA modification in cancer: mechanisms, tumor adaptation, and therapeutic vulnerabilities

**DOI:** 10.3389/fmolb.2026.1951991

**Published:** 2026-10-29

**Authors:** Xiaoxiao Tian, Di Huang, Chaonan Guan

**Affiliations:** 1 Department of Gastroenterology, The First Affiliated Hospital, College of Clinical Medicine, Henan University of Science and Technology, Luoyang, Henan, China; 2 Department of Child Healthcare, The Third Affiliated Hospital of Zhengzhou University, Zhengzhou, Henan, China; 3 Translational Medicine Center, The First Affiliated Hospital of Zhengzhou University, Zhengzhou, Henan, China

**Keywords:** cancer therapy, METTL1-WDR4, N7-methylguanosine, transfer RNA, translational control

## Abstract

N7-methylguanosine (m^7^G) is distributed across several RNA classes and regulates distinct stages of gene expression. The best-characterized form is the 5′cap of messenger RNA, although m^7^G also occurs in transfer RNA, ribosomal RNA, internal messenger RNA, circular RNA, and transfer RNA-derived small RNAs. Cancer-associated m^7^G dysregulation therefore cannot be ascribed to a single enzyme or molecular outcome. The strongest causal evidence concerns the METTL1-WDR4 complex, which modifies transfer RNA and reshapes codon-biased translation, promoting proliferation, metastasis, metabolic adaptation, stress tolerance, and treatment resistance. Studies of internal messenger RNA m^7^G report reader-dependent effects on stability, decay, translation, and stress-granule partitioning. Cap biochemistry is established, whereas catalytic cancer mechanisms involving ribosomal RNA remain less resolved. In preclinical models, these pathways connect tumor-cell adaptation with stromal communication, immune evasion, and treatment response. The biological effects of m^7^G vary with the modified RNA and the proteins favored by translation; tumor-restrictive activity emerges when altered tRNA modification increases the production of cell-cycle inhibitory proteins. This review organizes established and emerging mechanisms by RNA compartment and examines the substrate-dependent balance between tumor promotion, tumor restriction, and treatment sensitivity. It also evaluates biomarker strategies, emerging pharmacological and RNA-directed interventions, and the methodological standards needed to distinguish direct m^7^G-dependent effects from regulator expression correlations. A site-resolved, substrate-specific understanding of m^7^G may reveal translational dependencies that are actionable without disrupting essential RNA metabolism in normal tissues.

## 1 Introduction

Chemical modification expands the regulatory capacity of RNA beyond its primary sequence. Modified nucleotides can alter RNA folding, stability, localization, processing, translation, and interactions with RNA-binding proteins, thereby coupling gene expression to developmental, metabolic, and stress signals. In cancer, these processes influence oncogene output, tumor-suppressor activity, cellular plasticity, and responses to treatment, positioning the epitranscriptome alongside transcriptional and chromatin regulation as a determinant of malignant phenotype (Ranga et al., 2025).

Most cancer epitranscriptomic research initially centered on N6-methyladenosine, but a broader set of modifications is now linked to drug resistance and tumor evolution. This broader perspective matters because chemically distinct marks act on different RNA classes and control non-equivalent steps of gene expression (Tang et al., 2024). RNA methylation also participates in tumor immunity by modulating RNA metabolism, innate immune sensing, and immune-cell function, linking post-transcriptional regulation and the tumor microenvironment (Zhang et al., 2021). Clinical relevance extends to cytotoxic and targeted therapies, for which altered RNA-modifying proteins can change treatment sensitivity without requiring new DNA mutations (Wood et al., 2021). Reviews of cancer drug resistance now place N7-methylguanosine (m^7^G) among the RNA modifications capable of influencing RNA stability, translation, autophagy, invasion, and apoptosis (Chen D. et al., 2024).

m^7^G is unusual because the same chemical group is found in structurally and functionally distinct RNA contexts. The canonical m^7^G cap promotes the maturation and translation of RNA polymerase II transcripts, whereas internal m^7^G residues occur in transfer RNA (tRNA), ribosomal RNA (rRNA), messenger RNA (mRNA), and non-coding RNA. Cancer-focused studies have therefore shifted from cap-dependent translation toward tRNA modification, codon-biased translation, internal mRNA regulation, and modified small RNAs (Chen Y. et al., 2022). Gastrointestinal tumors provide a substantial evidence base linking METTL1-WDR4-dependent m^7^G to growth, metastasis, DNA repair, autophagy, and therapeutic resistance (Li et al., 2026). In hepatocellular carcinoma, m^7^G has also become part of a wider RNA methylation network associated with metabolic reprogramming and immune escape (Xu Y. et al., 2025).

Recent syntheses identify METTL1-WDR4-dependent tRNA methylation as the dominant experimental axis while distinguishing emerging internal mRNA and non-coding RNA mechanisms from the established biochemistry of cap methylation (Wu et al., 2025; Zhang et al., 2025b; Zhang et al., 2024). Broader assessments of m^7^G biology likewise connect the modification to translation, RNA stability, malignant progression, and treatment response (Cheng et al., 2022; Luo et al., 2022; Zhang X. et al., 2023). Disease-focused reviews emphasize that the same machinery can have distinct consequences in breast cancer and that immune effects require attention to both tumor cells and the microenvironment (Dai et al., 2024; Han et al., 2024). Recent immunotherapy studies also position METTL1-dependent tRNA m^7^G as a potential regulator of immune suppression and checkpoint response (Wang Q. et al., 2025).

Current evidence supports viewing m^7^G as a multilayered regulator of RNA fate rather than a uniform oncogenic mark. This review summarizes the molecular forms and detection of m^7^G, integrates RNA-compartment-specific mechanisms with cancer hallmarks and the tumor ecosystem, and evaluates the evidence linking m^7^G to therapeutic response. Particular emphasis is placed on causal validation, substrate- and cell-type-specific effects, biomarker design, and intervention strategies that distinguish modification-dependent biology from the broader functions of m^7^G-associated proteins. A central unresolved issue is why the same regulator can support oncogenic translation in one setting and cell-cycle restraint in another. The available data point to substrate selection, rather than total modification abundance, as a likely source of this divergence.

## 2 The molecular basis of m^7^G regulation

m^7^G biology is determined by the location of the modified guanosine, the enzyme complex that deposits it, and the proteins that interpret the resulting RNA state. These variables separate cap-dependent translation from tRNA stability, rRNA maturation, internal mRNA fate, and modified non-coding RNA function. A compartment-resolved map of substrates, regulators, and detection methods provides a basis for comparing cancer mechanisms without treating all m^7^G signals as equivalent (Figure 1). Table 1 compares the sites, writer complexes, effectors, and cancer-evidence boundaries across these compartments.

**FIGURE 1:**
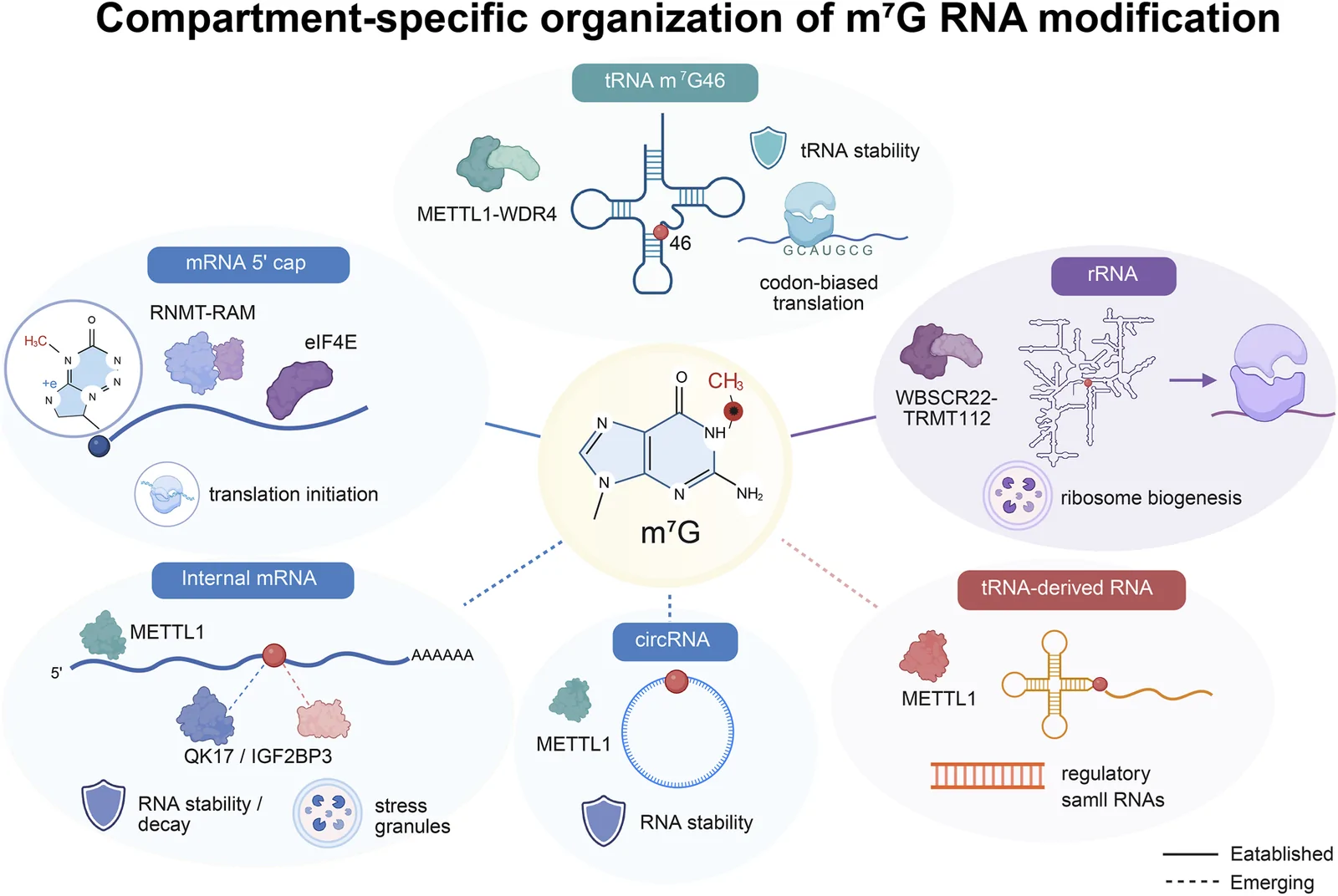
Compartment-specific organization of m^7^G RNA modification. This figure distinguishes the mRNA 5′cap, tRNA m^7^G46, rRNA, internal mRNA, circRNA, and tRNA-derived RNA layers and links each to its principal writer, reader or effector, RNA-fate consequence, and level of evidence. Solid arrows indicate established catalytic relationships, whereas dashed arrows indicate emerging or incompletely validated pathways. Abbreviations: circRNA, circular RNA; m^7^G, N7-methylguanosine; mRNA, messenger RNA; rRNA, ribosomal RNA; tRNA, transfer RNA. Created in BioRender. Guan (2026) https://BioRender.com/gddt3uf.

**TABLE 1:** Compartment-resolved biochemical mechanisms and cancer-evidence boundaries.

RNA compartment/site	Writer/cofactor	Reader or effector	Biological consequence	Cancer evidence and causal boundary	Ref.
mRNA 5′cap; terminal guanosine	RNMT/RAM	CBC; eIF4E; eIF4E3	Processing, export, protection, translation initiation	Cap chemistry is established; genotype-selective RNMT dependency and cap-reader phenotypes define the current cancer evidence base	(Borden et al., 2021; Dunn and Lombardi and Lukoszek and Cowling, 2019; Mars and Ghram and Culjkovic-Kraljacic and Borden, 2021; Osborne et al., 2013; Xie et al., 2023)
Cytoplasmic tRNA; G46 in a subset of tRNAs	METTL1/WDR4	No dedicated reader established; functional output is mediated through tRNA structural stability and cognate codon decoding	tRNA maintenance and codon-biased translation	Strongest catalytic and cancer evidence among non-cap m^7^G pathways; target proteins and phenotype vary among tumor types	(Chen et al., 2022b; Dai et al., 2021; Du et al., 2024; Li et al., 2023a; Orellana et al., 2021)
Human 18 S rRNA; G1639	WBSCR22/BUD23/ TRMT112	No dedicated reader has been established	Modification and small-subunit maturation are separable functions	The site assignment is established; cancer studies link pathway components to growth and drug phenotypes, with catalytic and non-catalytic functions considered separately	(Chi et al., 2020; Haag and Kretschmer and Bohnsack, 2015; Khan et al., 2022; Zorbas et al., 2015)
Internal mRNA; reported UTR or transcript-body sites	METTL1-associated in selected studies; writer assignment is model dependent	QKI7/G3BP1; IGF2BP3; other effectors vary	Reader-dependent sequestration, decay, or reported stabilization	Site-directed reader evidence accompanies enrichment-based target assignments; the degree of site resolution and writer attribution varies among models	(Li et al., 2023b; Liu et al., 2024; Zhang et al., 2023a; Zhao et al., 2023)
circRNA; reported internal sites	METTL1 or TRMT10C (reported in a single model) assigned in individual models; cofactor context varies	No conserved reader established; model-specific interacting proteins	Reported stability changes and scaffold output	circKDM1A site mutation supports a local mechanism; writer attribution is evaluated at model level	(Guo et al., 2024; Sun et al., 2024; Zhang et al., 2026a; Zhao et al., 2024)
tRNA-derived RNA; retained parental site only when demonstrated	Parental tRNA modified by METTL1/WDR4; cleavage creates the fragment	ANXA2 in the LysTTT fragment example	Fragment abundance or modified-fragment signaling; translation programs in prostate cancer	Separate regulation of cleavage from direct action of a mark retained within a fragment	(García-Vílchez et al., 2023a; García-Vílchez et al., 2023b; Ying et al., 2024)

### 2.1 RNA compartments and molecular consequences

The 5′m^7^G cap is installed on nascent RNA polymerase II transcripts and supports protection from exonucleases, RNA processing, nuclear export, and translation. Capping is not purely constitutive: transcript capping and recapping can vary with cellular state, and elevated capping of selected growth- and survival-related transcripts has been observed in primary cancer material and experimental models with increased RNA guanine-7 methyltransferase (RNMT), MYC, or eIF4E activity (Borden et al., 2021). The cap therefore serves as both a structural requirement and a regulated gate for transcript competence. Unlike an internal modified nucleotide, the cap guanosine is linked to the transcript through a 5′–5′ triphosphate bridge. RNMT acts during nascent transcript processing, creating a binding platform for cap-binding proteins rather than modifying a nucleotide within the RNA body.

The cap-binding protein eukaryotic translation initiation factor 4E (eIF4E) recruits capped mRNAs to the translation-initiation machinery and also participates in the nuclear export of selected transcripts. Its abundance and activity can preferentially enhance the expression of growth-promoting mRNAs, establishing cap recognition as an oncogenic translational node (Piserà et al., 2018). Internal m^7^G has a different topology and cannot recruit the canonical cap complex in the same manner. Instead, its effects depend on local RNA structure, transcript-specific binding proteins, and the position of the modified nucleotide.

Cap-associated regulation is divided between the nuclear cap-binding complex (CBC, comprising NCBP1/CBP80 and NCBP2/CBP20) and cytoplasmic eIF4E-containing complexes, which coordinate RNA maturation, export, surveillance, and translation (Mars et al., 2021). Nuclear cap-binding proteins and cap hypermethylation can also preserve selected RNA outputs when canonical eIF4E-dependent translation is constrained, adding a second layer of cap-dependent selectivity (Boris-Lawrie et al., 2025). Cap-dependent initiation therefore differs from the codon-decoding effects of tRNA m^7^G and the reader-dependent fate of internal mRNA sites. Increased translation is a shared output, not evidence of a shared biochemical mechanism.

tRNA m^7^G is concentrated at guanosine 46 in the variable loop of a subset of cytoplasmic tRNAs. This position stabilizes tRNA architecture and supports a functional pool of mature tRNAs capable of decoding codons during translation. Changes in the abundance or modification of particular tRNAs can favor proteins encoded by transcripts enriched for their cognate codons, thereby producing codon-biased translation rather than a uniform increase in protein synthesis (Dedon and Begley, 2022). rRNA m^7^G is associated with ribosome maturation, although writer-dependent maturation and catalytic methylation must be distinguished, whereas m^7^G signals in circular RNAs and tRNA-derived fragments can influence RNA stability or acquire functions that are not reducible to the parental RNA (Cai et al., 2023).

### 2.2 Writers, cofactors, and readers

RNMT, activated by the RNMT-activating miniprotein (RAM), catalyzes N7 methylation of the guanosine cap. Molecular simulations indicate that RAM stabilizes RNMT conformations favorable for cap and S-adenosylmethionine binding, offering a mechanistic basis for allosteric activation and a structural starting point for inhibitor development (Bueren-Calabuig et al., 2019). The RNMT-RAM axis is therefore mechanistically distinct from internal m^7^G writer complexes.

METTL1 and WD repeat domain 4 (WDR4) form the best-characterized tRNA m^7^G methyltransferase. Structural and biochemical analyses show that WDR4 scaffolds METTL1 and the tRNA T-arm, whereas METTL1 regions surrounding the catalytic pocket secure the variable loop; phosphorylation of METTL1 at serine 27 disrupts the catalytic center and inhibits methylation (Li J. et al., 2023). Together, these observations define substrate recognition and post-translational control at atomic resolution. WBSCR22 (also called BUD23), with TRMT112, contributes to rRNA modification and small-subunit biogenesis, but direct evidence connecting its catalytic m^7^G activity to cancer is less developed than that for METTL1-WDR4 (Haag et al., 2015; Zorbas et al., 2015).

Readers and effectors are also substrate dependent. eIF4E recognizes the terminal m^7^G cap, whereas Quaking proteins and insulin-like growth factor 2 mRNA-binding proteins have been implicated in the interpretation of internal mRNA m^7^G. No physiological m^7^G eraser has achieved broad experimental acceptance. Accordingly, applying a symmetric writer-reader-eraser model to m^7^G would imply an unestablished reversibility.

Phosphorylation further alters cap-reader behavior. Cancer-focused evidence links phosphorylation of eIF4E to selective translation, invasion, and therapeutic adaptation (Yang et al., 2020). Molecular modeling suggests that phosphorylation at serine 209 reorganizes the cap-binding interface and changes electrostatic compatibility with capped RNA, offering a structural explanation for signal-dependent reader activity (Lama and Verma, 2020).

### 2.3 Detection and compartment assignment

Reliable m^7^G analysis must distinguish terminal caps from internal residues and separate mRNA from abundant tRNA and rRNA. m^7^G-quant-seq uses chemical reduction and depurination to convert internal m^7^G into abasic sites that produce characteristic reverse-transcription variations, enabling single-base quantification of m^7^G fractions in human cytoplasmic tRNAs (Zhang et al., 2022). Borohydride reduction sequencing similarly supports base-resolution mapping, whereas antibody-based measurements can compare relative m^7^G abundance but remain vulnerable to batch-dependent antibody performance (D'Ambrosi et al., 2024).

Live-cell interaction assays can complement chemical mapping by measuring reader engagement rather than modification abundance. CRISPR RiPCA has been adapted to quantify eIF4E binding to m^7^G-capped RNA and to assess on-target disruption of this interaction by candidate inhibitors (Vega-Hernández et al., 2025).

#### 2.3.1 Experimental standards for establishing m^7^G causality

No single assay establishes that an individual RNA site causes a cancer phenotype. A causal assignment requires connecting writer perturbation to modification of a defined substrate, altered RNA fate, and phenotypic rescue. Wild-type rescue should be compared with an expression- and localization-matched catalytic-dead writer, because protein depletion can also alter scaffolding, binding, or secondary transcriptional programs. Internal mRNA studies additionally require explicit exclusion of cap-derived signal and abundant structured RNAs. These standards are particularly important when methylated RNA immunoprecipitation is used to nominate a transcript but the functional experiment manipulates the writer globally. For a proposed writer, substrate binding must be distinguished from methyl transfer using purified-protein assays and an appropriate unmodified RNA substrate. Site substitution or targeted RNA manipulation should control for changes in sequence, structure, and reader recruitment. Reader depletion and binding-defective rescue can test whether a nominated effector mediates the RNA-fate change. Transcript half-life measurements address stability; polysome or ribosome profiling addresses translation. tRNA mechanisms additionally require cognate tRNA measurements and codon-dependent reporters or recoding. These complementary tests establish different links in a causal chain; no single global m^7^G signal can replace them.

### 2.4 RNA homeostasis and malignant adaptation

Cancer cells place unusually high and variable demands on RNA processing and protein synthesis. m^7^G can address these demands at several levels: cap methylation licenses transcript processing and translation, tRNA modification preserves decoding capacity, rRNA modification supports ribosome production, and internal m^7^G has been linked to the persistence or subcellular handling of selected transcripts. METTL1-WDR4 and QKI illustrate how m^7^G-associated writers and readers can control RNA stability and translation in digestive diseases, although their effects depend on RNA class and cell type (Zhou et al., 2024).

This layered organization suggests that tumors may become dependent on m^7^G through different routes. A rapidly proliferating tumor may rely on codon-matched tRNAs, whereas a treated tumor may use internal mRNA regulation or protected tRNA pools to survive stress. The actionable dependency is therefore not high m^7^G *per se*, but a defined enzyme-substrate-effector circuit operating in a specific cellular state.

## 3 RNA-specific mechanisms in cancer

Aberrant m^7^G regulation originates from transcriptional activation, altered protein stability, oncogenic signaling, or treatment-induced selection. These inputs reshape tRNA decoding, internal mRNA fate, cap-dependent translation, and non-coding RNA output. These circuits converge on proliferation, invasion, metabolism, stress tolerance, and immune interactions, but the direction and magnitude of the phenotype depend on which transcripts gain translational or stability advantages (Figure 2; Table 2).

**FIGURE 2:**
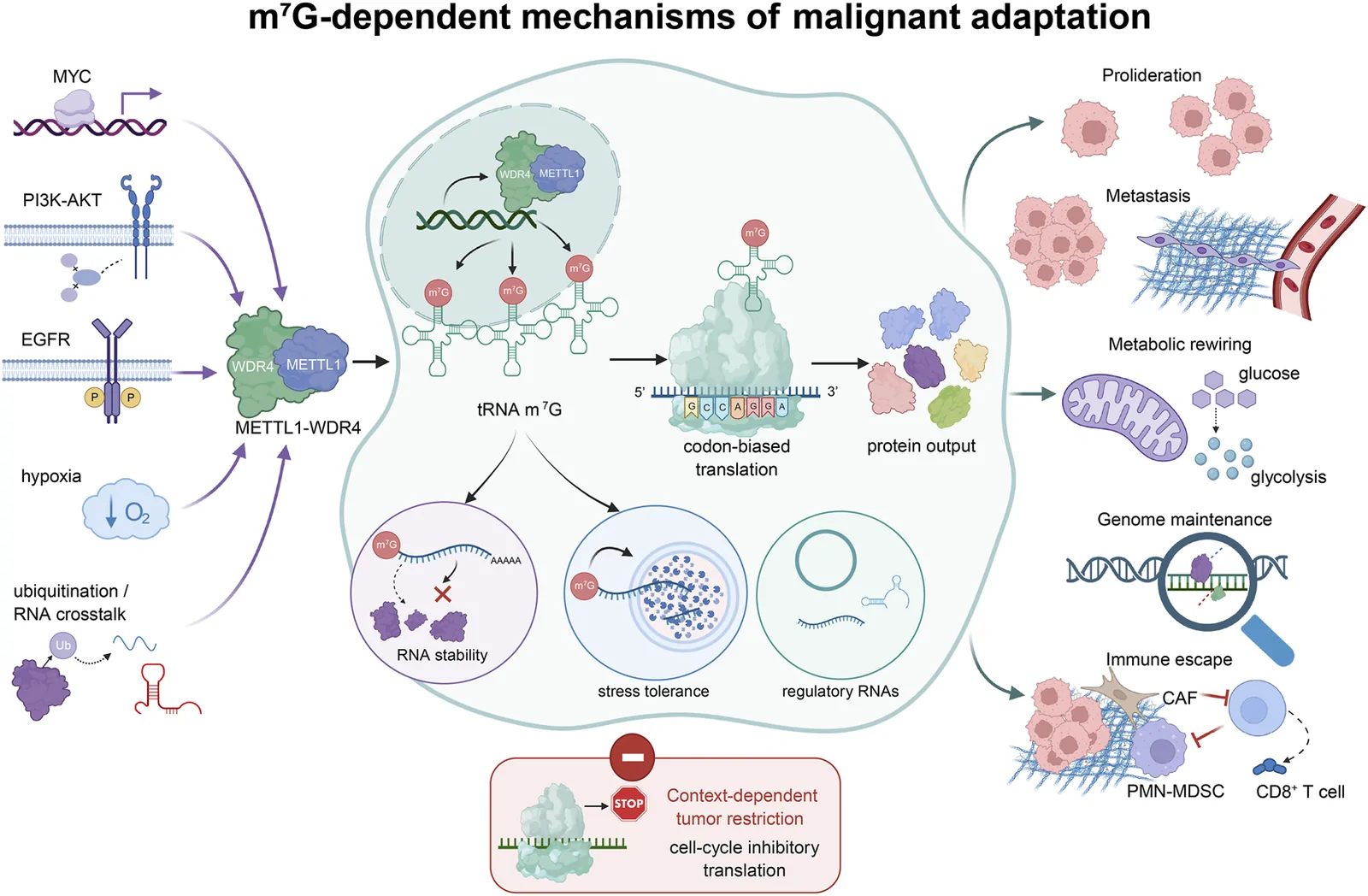
m^7^G-associated RNA mechanisms shaping tumor adaptation and restriction. Upstream transcriptional and post-translational signals alter m^7^G machinery, which then controls codon-biased translation, transcript stability or degradation, stress-granule partitioning, and regulatory RNA production. These RNA outcomes converge on proliferation, metastasis, metabolism, stress responses, and immune escape. Tumor-cell-intrinsic programs alter immune signaling through cytokines, metabolites, or surface ligands, whereas immune-cell-intrinsic mechanisms require perturbation within the immune compartment; m^7^G-independent functions of associated proteins should be displayed separately. Created in BioRender.

**TABLE 2:** Molecular mechanisms linking m^7^G regulation to cancer phenotypes. Entries are organized by RNA mechanism; the causal-status column distinguishes direct m^7^G-dependent evidence from regulator-associated or m^7^G-independent phenotypes. The evidence-basis column summarizes the reported model systems.

RNA mechanism	Causal status	Cancer type	Regulator or RNA layer	Target or mechanism	Core finding	Evidence basis	Ref.
Writer regulation (substrate unresolved)	m^7^G-associated	HCC	MYC-WDR4	CCNB1 translation; PI3K/AKT signaling	Promotes growth, metastasis, and sorafenib resistance	Clinical samples; cells; xenografts	(Xia et al., 2021)
Internal mRNA fate	m^7^G-associated	Endometrial cancer	CBLC-METTL1	METTL1 ubiquitination; ESRRA mRNA stabilization	Writer stabilization promotes malignant progression	Single-cell data; cells; xenografts	(Peng and Zhang and Wang and Wang and Liu, 2026)
Internal mRNA fate	m^7^G-associated	HNSCC; digestive cancers	METTL3-METTL1	m6A-METTL1 regulation; CDK4 m^7^G stabilization	Links m6A signaling to m^7^G-dependent proliferation	Patient data; cancer cell models	(Zhang and Cui, 2024)
Writer regulation (substrate unresolved)	m^7^G-associated	Esophageal cancer	NSUN5-METTL1	m5C modification of METTL1 mRNA	Upstream m5C increases METTL1 and tumor growth	Clinical samples; cells; xenografts	(Cui and Hu and Zhang, 2025)
Writer regulation (substrate unresolved)	m^7^G-associated	Gastric cancer	TSPAN31-METTL1-CCT2	Co-expression-associated growth signaling	Supports proliferation and migration in an METTL1-associated regulatory context	Clinical samples; cell assays	(Ma et al., 2022)
tRNA decoding	Direct m^7^G-dependent	Pan-cancer transformation	METTL1-tRNA m^7^G	Arg-TCT-4–1; AGA-biased translation	Codon-biased translation drives oncogenic transformation	Multi-omics; cells; mouse tumors	(Orellana et al., 2021)
tRNA decoding	Direct m^7^G-dependent	ICC	METTL1-WDR4	Cell-cycle and EGFR-pathway translation	tRNA m^7^G promotes tumorigenesis and progression	Clinical samples; cells; mouse models	(Dai et al., 2021)
tRNA decoding	Direct m^7^G-dependent	HNSCC	METTL1-WDR4	PI3K/AKT/mTOR-biased translation	Drives progression and alters tumor-stromal interactions	Clinical samples; cells; transgenic mice	(Chen et al., 2022b)
tRNA decoding	Direct m^7^G-dependent	Papillary thyroid cancer	METTL1-tRNA m^7^G	Codon-specific TNF-α translation	Promotes proliferation and metastasis	Clinical samples; cells; xenografts	(Li et al., 2025b)
tRNA decoding	Direct m^7^G-dependent	Lung cancer	METTL1-WDR4	m^7^G-tRNA-decoded codon translation	Supports growth and invasion through codon usage	Clinical samples; cells; xenografts	(Ma et al., 2021)
tRNA decoding	Direct m^7^G-dependent	Bladder cancer	METTL1 plus METTL3	tRNA m^7^G and mRNA m6A enhance TROP2 translation	Dual RNA marks cooperate in tumor progression	Clinical samples; cells; xenografts	(Chen et al., 2023)
tRNA decoding	Direct m^7^G-dependent	Breast cancer	METTL1-tRNA m^7^G	GADD45 A and RB1 codon-biased translation	Restricts growth and enhances CDK4/6 inhibition	Clinical samples; multi-omics; xenografts	(Du et al., 2024)
tRNA decoding	Direct m^7^G-dependent	Bladder cancer	METTL1-tRNA m^7^G	EGFR and EFEMP1 translation	Promotes proliferation, migration, and invasion	Clinical samples; multi-omics; xenografts	(Ying et al., 2021)
tRNA decoding	m^7^G-associated	Prostate cancer	METTL1-WDR4	PI3K/AKT/mTOR; MYC and VEGF	Supports oncogenic phenotypes; model-limited evidence	Patient samples; DU145 cells	(Deng et al., 2025a)
tRNA decoding	Direct m^7^G-dependent	CRC liver metastasis	METTL1-tRNA m^7^G	CCND3 and PI3K/AKT translation	Catalytic activity promotes liver metastasis	Proteomics; single-cell; cells; mice	(Chen et al., 2026)
tRNA decoding	Direct m^7^G-dependent	Gastric cancer	METTL1-WDR4 tRNA m^7^G	SDHAF4 translation; respiratory complex II	Increases oxidative phosphorylation and progression	Clinical samples; multi-omics; mice	(Xu et al., 2025a)
tRNA decoding	Direct m^7^G-dependent	ESCC	METTL1-WDR4 tRNA m^7^G	RPTOR-ULK1-autophagy translation	Maintains tumor-promoting autophagy control	Clinical samples; cells; transgenic mice	(Han et al., 2022)
tRNA decoding	Direct m^7^G-dependent	Multiple cancers	METTL1-tRNA m^7^G	Protection from stress-induced tRNA cleavage	Preserves stress survival and tumor growth	Cells; mouse tumors	(García-Vílchez et al., 2023b)
Internal mRNA fate	Direct m^7^G-dependent	Cancer stress response	QKI7 reader	m^7^G RNA transport to G3BP1 stress granules	Alters RNA stability, translation, and chemosensitivity	Transcriptomics; cell assays	(Zhao et al., 2023)
Internal mRNA fate	Direct m^7^G-dependent	Glioblastoma	IGF2BP3 reader	Internal m^7^G-dependent TP53 mRNA degradation	Reader-controlled decay alters progression and drug response	Site targeting; cells; xenografts	(Liu et al., 2024)
Internal mRNA fate	m^7^G-associated	CRPC	P300-SP1-METTL1	Internal m^7^G stabilizes CDK14 mRNA	Promotes proliferation and invasion	Clinical samples; multi-omics; xenografts	(Zhang et al., 2023a)
Internal mRNA fate	m^7^G-associated	OSCC	METTL1	5′UTR m^7^G stabilizes NEK1 mRNA	Supports proliferation and G1-S progression	Clinical samples; cells; xenografts	(Chen et al., 2025)
Internal mRNA fate	m^7^G-associated	CRC	METTL1	Internal m^7^G stabilizes MACC1 and SDCCAG8	Drives growth through two rescued targets	Clinical samples; cells; xenografts	(Yu and Bao and Li and Gao and Chen, 2026)
Internal mRNA fate	m^7^G-associated	CRC	METTL1	m^7^G-dependent RRP9 mRNA stabilization	Activates PI3K/AKT and stem-like growth	Clinical samples; cells; xenografts	(Li and Jing and Xu and Wang, 2025a)
Internal mRNA fate	m^7^G-associated	OSCC	Internal mRNA m^7^G	BIRC3-associated m^7^G methylome	Maps altered m^7^G patterns across disease transition	Clinical samples; MeRIP-seq; cells	(Li et al., 2023b)
Internal mRNA fate	m^7^G-associated	HBV-associated HCC	Internal mRNA m^7^G	EZH2, SMARCA4, and YY1 modification	Defines dynamic modification during hepatocarcinogenesis	Public methylome; *in vitro* validation	(Xiao et al., 2025b)
Internal mRNA fate	m^7^G-associated	Gastric cancer	METTL1-WDR4	PIK3C2B and AKT mRNA stabilization	YY1-linked feedback promotes migration and growth	Clinical samples; cells; xenografts	(Wang et al., 2025b)
Internal mRNA fate	m^7^G-associated	Metastatic CRC	METTL1	m^7^G-dependent ICAM1 mRNA stabilization	Enhances adhesion-associated metastatic behavior	Patient data; cells; mouse models	(Cai et al., 2025)
Internal mRNA fate	m^7^G-associated	Glioma	METTL1-internal m^7^G	PGK1 mRNA stabilization	Enhances glycolysis and tumor growth	Clinical samples; cells; xenografts	(Fan and Wang and Qin and Yan and Du, 2025)
Internal mRNA fate	m^7^G-associated	HCC	METTL1-internal m^7^G	ASNS mRNA stabilization; mTOR activation	Reprograms asparagine metabolism	Multi-omics; cells; mouse models	(Xing et al., 2026)
Internal mRNA fate	m^7^G-associated	CRC microenvironment	CAF exosomes-METTL1	SLC1A5 mRNA stabilization	Increases glutamine metabolism and metastasis	CAF-conditioned cells; xenografts	(Zhang et al., 2026b)
Internal mRNA fate	m^7^G-associated	Cutaneous SCC	METTL1-internal m^7^G	ATF4 mRNA stabilization	Promotes glycolysis and malignant progression	Clinical samples; cells; xenografts	(Zhang et al., 2025a)
Internal mRNA fate	m^7^G-associated	CRC	P300-ETV4-WDR4	SPP1 mRNA stabilization; autophagy suppression	Couples chromatin activation to m^7^G signaling	Patient data; cells; xenografts	(Deng and Xie and Gong, 2025b)
Internal mRNA fate	m^7^G-associated	CRC	METTL1-PKM2	Glycolysis; H3K9 lactylation; CD155 transcription	Promotes immune escape through metabolic feedback	Clinical samples; cells; mice	(Wang et al., 2024)
Regulatory ncRNA	Direct m^7^G-dependent	Prostate cancer	METTL1-tRNA m^7^G	5′tRNA-derived small RNA biogenesis	Reprograms translation and antitumor immunity	Clinical samples; multi-omics; mouse models	(García-Vílchez et al., 2023a)
Regulatory ncRNA	Direct m^7^G-dependent	Bladder cancer	m^7^G-3′ tiRNA LysTTT	ANXA2 Tyr24 phosphorylation	Modified tiRNA promotes malignant behavior	Carcinogenesis models; cells; mice	(Ying et al., 2024)
Regulatory ncRNA	Direct m^7^G-dependent	CRC	METTL1-circKDM1A	m^7^G-dependent circRNA stabilization; AKT activation	Modified circRNA drives growth and invasion	Site mutation; cells; xenografts	(Sun et al., 2024)
Regulatory ncRNA	m^7^G-associated	CRC	circ_0003323-METTL1	KLK6 m^7^G-associated regulation	circRNA and writer cooperate in tumor progression	Cells; mouse xenografts	(Zhang et al., 2026a)
Regulatory ncRNA	m^7^G-associated	Laryngeal cancer	WDR4-ZFAS1-RBFOX2	m^7^G-dependent lncRNA stabilization and feedback	Feedback circuit promotes growth and invasion	Clinical samples; cells; xenografts	(Lu et al., 2025)
Regulatory ncRNA	m^7^G-associated	HCC	METTL1-circIPP2A2	Hornerin-PI3K scaffold; AKT-GSK3β signaling	m^7^G-stabilized circRNA promotes progression	Clinical samples; cells; xenografts	(Guo et al., 2024)
Regulatory ncRNA	m^7^G-associated	Bladder cancer	METTL1-miR-760	m^7^G-dependent miRNA processing; ATF3 repression	Modified miRNA pathway drives progression	Multi-omics; cells; xenografts	(Xie et al., 2022)
Regulatory ncRNA	m^7^G-associated	Lung cancer	TRMT10C-circFAM126 A	TRMT10C-associated m^7^G enrichment and circRNA stabilization	Stabilized circRNA restrains glycolytic tumor growth in the reported TRMT10C-associated model	Clinical samples; cells; xenografts	(Zhao et al., 2024)
Cap-dependent regulation	Direct cap mechanism	PIK3CA-mutant breast cancer	RNMT-cap m^7^G	PI3Kα-dependent cap-methylation addiction	Oncogenic genotype creates selective RNMT dependency	Cancer and normal mammary cells	(Dunn and Lombardi and Lukoszek and Cowling, 2019)
Cap-dependent regulation	Direct cap mechanism	MYC-driven cancer	MYC-RNMT	Cap methylation of Wnt pathway transcripts	MYC increases Wnt output beyond transcription	Promoter and translation assays	(Posternak and Ung and Cheng and Cole, 2017)
Cap-dependent regulation	m^7^G-associated	Glioma stem-like cells	B7-H6-c-MYC-RNMT	RNMT-associated proliferative signaling	Links immune ligand signaling to RNMT expression	Patient data; stem-like cell models	(Chen et al., 2020)
Cap-dependent regulation	Direct cap mechanism	PDAC	NCBP2 cap reader	c-JUN translation; MEK-ERK activation	Cap reader promotes pancreatic tumor growth	Patient cohorts; cells; xenografts	(Xie et al., 2023)
Cap-dependent regulation	Direct cap mechanism	Breast, colon, liver, lung cancers	CBP20 cap reader	Cell-cycle transcript regulation	Reader depletion induces arrest and apoptosis	Pan-cancer data; cells; compound screening	(Cho et al., 2025)
Cap-dependent regulation	Direct cap mechanism	High-eIF4E cancers	eIF4E3 cap reader	Atypical m^7^G-cap recognition	Competes with eIF4E and suppresses transformation	Structural; cellular; patient data	(Osborne et al., 2013)
Cap-dependent regulation	Direct cap mechanism	Breast cancer metastasis	Phosphorylated eIF4E	SNAIL and MMP3 translation	Cap-reader phosphorylation enables EMT and metastasis	Cells; genetically engineered mice	(Robichaud et al., 2015)
rRNA-associated regulation	m^7^G-associated	Clear-cell renal cancer	METTL1 and BUD23	tRNA/rRNA writers; tumor-suppressor transcripts	Both writers support oncogenic phenotypes	Clinical samples; cells	(Su et al., 2025)
rRNA-associated regulation	m^7^G-associated	Glioma	WBSCR22	AKT-GSK3β-β-catenin signaling	Promotes survival and invasion in a WDR4-associated regulatory program	Patient data; cell assays	(Chi et al., 2020)
rRNA-associated regulation	m^7^G-associated	Pancreatic cancer	WBSCR22-TRMT112	Transcriptional repression of ISG15	Writer complex shows tumor-restrictive activity	Cells; mouse xenografts	(Khan et al., 2022)
Regulator function (substrate unresolved)	m^7^G-associated	Glioma	WDR4	PI3K/AKT-CDK1/2 signaling	Promotes proliferation and cell-cycle progression	Public cohorts; cells; xenografts	(Liu et al., 2025b)
Regulator function (substrate unresolved)	m^7^G-associated	CRC	METTL1	CHEK2-dependent G1-S arrest	METTL1 relieves checkpoint-mediated growth arrest	CRC cell models	(Jiang et al., 2024)
Regulator function (substrate unresolved)	m^7^G-associated	CRC	WDR4	GSK3β binding; β-catenin activation	Promotes growth and invasion through protein-binding and signaling activity	Clinical samples; cells; xenografts	(Wang et al., 2025a)
Regulator function (substrate unresolved)	m^7^G-associated	Bladder cancer	WDR4-DDX20-EGR1	Transcriptional repression of ARRB2	Drives lymphatic metastasis independent of proven m^7^G	Clinical samples; cells; xenografts	(Wang et al., 2023a)
Regulator function (substrate unresolved)	m^7^G-associated	CRC	GEMIN5-SHMT2	m^7^G-associated mRNA stabilization; Wnt signaling	Suppresses ferroptosis; writer assignment uncertain	Clinical samples; cell assays	(Wang and Wu and He and Zhu and Li, 2025d)
Regulator function (substrate unresolved)	m^7^G-associated	Breast cancer	WDR4	mTORC1 and immune-suppressive state	Supports tumor growth and immunotherapy resistance	Bulk and single-cell data; cells; mice	(Luo et al., 2025)
m^7^G-independent protein function	m^7^G-independent	HCC macrophages	WDR4-eIF4E2	ABCA1 translation; cholesterol efflux	Reprograms macrophages independently of m^7^G	Clinical samples; myeloid models; mice	(Liu et al., 2025a)

### 3.1 Upstream activation of m^7^G machinery

Oncogenic pathways can increase m^7^G machinery before changes in RNA substrates are measured. In hepatocellular carcinoma (HCC), MYC activates WDR4 transcription, and elevated WDR4 enhances CCNB1 expression, cell-cycle progression, epithelial-mesenchymal transition, and sorafenib resistance through a pathway connected to PI3K/AKT and p53 signaling (Xia et al., 2021). This mechanism links a canonical oncogenic transcription factor to the translational apparatus, while also illustrating the need to distinguish WDR4-dependent RNA regulation from direct site-resolved m^7^G effects.

Writer abundance is also regulated post-translationally. In endometrial cancer, the E3 ubiquitin ligase CBLC stabilizes METTL1 through ubiquitination, increases m^7^G modification and stability of ESRRA mRNA, and promotes proliferation, migration, and invasion (Peng et al., 2026). Ubiquitination therefore need not mark an m^7^G writer for degradation; it can increase writer persistence and connect proteostasis to epitranscriptomic output. Thus, transcriptional induction and protein stabilization provide distinct routes by which tumors can raise m^7^G capacity.

Cross-modification and co-expression networks provide additional upstream control. In head and neck and digestive-system cancer models, METTL3-dependent m6A increases METTL1 expression, after which METTL1 deposits m^7^G on CDK4 mRNA and promotes cell-cycle progression (Zhang and Cui, 2024). In esophageal cancer, NSUN5 binds METTL1 mRNA and increases its m5C modification and expression, creating a sequential m5C-to-m^7^G regulatory axis with functional rescue in xenografts (Cui et al., 2025). Gastric cancer data additionally place TSPAN31, METTL1, and CCT2 in a pro-proliferative co-expression pathway, although direct m^7^G dependence was not established (Ma et al., 2022). Altered regulator abundance identifies upstream control of the pathway but does not reveal the relevant RNA substrate or catalytic output. Transcriptional, proteostatic, and cross-modification inputs therefore provide different levels of evidence for an m^7^G-driven phenotype.

### 3.2 tRNA m^7^G and codon-biased translation

The most complete causal model involves METTL1-dependent modification of tRNA. METTL1 amplification or overexpression increases m^7^G-modified tRNAs, particularly Arg-TCT-4-1, and favors translation of AGA codon-enriched cell-cycle regulators; expression of this single tRNA is sufficient to promote oncogenic transformation in experimental systems (Orellana et al., 2021). This finding shows that a tRNA modification can select a translational program through codon content rather than globally accelerating protein synthesis.

Tissue-defined cancer models provide direct tests of codon-biased translation. In intrahepatic cholangiocarcinoma (ICC), METTL1 and WDR4 support tumor growth in cell, xenograft, knockout, and overexpression models by promoting translation of cell-cycle and epidermal growth factor receptor pathway transcripts enriched for m^7^G-tRNA-decoded codons (Dai et al., 2021). In head and neck squamous cell carcinoma, loss of METTL1 reduces m^7^G on 16 tRNAs, suppresses translation of PI3K/AKT/mTOR pathway transcripts, and changes tumor-stromal interactions in genetically engineered mouse models (Chen J. et al., 2022). Papillary thyroid cancer provides a more target-focused example in which catalytic METTL1 activity maintains specific m^7^G tRNAs and enhances codon-dependent translation of tumor necrosis factor-α, thereby supporting proliferation and metastasis (Li W. et al., 2025).

Lung cancer independently confirms that METTL1-WDR4 controls translation according to the frequency of codons decoded by m^7^G-modified tRNAs, with catalytic perturbation reducing tumor growth and invasion *in vivo* (Ma et al., 2021). Bladder cancer reveals cooperation between RNA marks: METTL1-dependent tRNA m^7^G and METTL3-dependent mRNA m6A jointly increase TROP2 translation, and dual writer depletion produces stronger antitumor effects than either perturbation alone (Chen et al., 2023).

These studies support a shared mechanism with variable downstream outputs. The modified tRNA repertoire defines which codon-biased transcripts benefit, while the tissue-specific transcriptome determines whether the dominant products regulate cell cycle, growth-factor signaling, inflammation, or another phenotype. Codon content is therefore a mechanistic variable, not merely a sequence feature. The conserved feature is substrate-dependent decoding, whereas the direction of tumor growth depends on the proteins favored by that decoding program. The breast cancer findings therefore refine the biological outcome of codon-biased translation without contradicting the decoding mechanism.

### 3.3 Internal mRNA m^7^G and RNA fate

Internal mRNA m^7^G introduces a reader-dependent layer of transcript control. Quaking proteins preferentially bind internal m^7^G-marked mRNAs, and the QKI7 isoform interacts with G3BP1 to move these transcripts into stress granules, changing their stability and translation while increasing cancer-cell sensitivity to chemotherapy through reduced Hippo-pathway translation (Zhao et al., 2023). By contrast, IGF2BP family proteins can recognize internal m^7^G, with IGF2BP3 promoting degradation of modified targets; targeted recruitment of METTL1 or IGF2BP3 to an m^7^G-containing region of the TP53 3′untranslated region altered transcript half-life, glioblastoma progression, and chemosensitivity (Liu et al., 2024). Internal m^7^G can therefore support either sequestration or decay, depending on reader identity and cellular state.

Cancer studies increasingly attribute transcript-specific stabilizing effects to METTL1. In castration-resistant prostate cancer, a P300-SP1 complex increases METTL1 transcription, and METTL1-associated internal m^7^G was reported to stabilize CDK14 mRNA to support proliferation and invasion (Zhang M. et al., 2023). In oral squamous cell carcinoma, METTL1-associated m^7^G in the 5′untranslated region of NEK1 mRNA was reported to prolong transcript stability and promote G1-S progression in cell and xenograft models (Chen et al., 2025). A colorectal cancer study similarly identified MACC1 and SDCCAG8 as internal m^7^G targets whose stabilization rescued growth defects caused by METTL1 depletion (Yu et al., 2026).

Additional evidence in colorectal cancer links METTL1-mediated m^7^G to stabilization of RRP9 mRNA, activation of PI3K/AKT signaling, stem-like growth, and xenograft expansion (Li N. et al., 2025). Transcriptome-wide mapping in oral squamous cell carcinoma identified thousands of internal m^7^G peaks and nominated BIRC3 as a modified pro-invasive transcript, but functional experiments tested BIRC3 rather than the causal requirement for its individual m^7^G sites (Li M. et al., 2023). Reanalysis and experimental validation across hepatitis B virus infection and HCC similarly identified dynamic internal m^7^G changes in EZH2, SMARCA4, and YY1, providing a disease-transition map rather than a completed writer-target mechanism (Xiao Y. et al., 2025).

The coexistence of m^7^G-linked stabilization and degradation is biologically plausible because a modification does not determine RNA fate independently of readers and local structure. Nevertheless, direct internal mRNA assignments warrant greater caution than tRNA m^7^G46 because immunoprecipitation-based signals can reflect antibody behavior, cap contamination, or indirect changes in RNA abundance. Enrichment peaks nominate RNA regions and do not establish single-nucleotide occupancy or stoichiometry. Agreement between enrichment and base-resolution chemical mapping should be tested on purified, decapped mRNA with input normalization and modified and unmodified controls. Bulk measurements cannot distinguish low occupancy in many transcripts from high occupancy in a minor RNA population. METTL1 involvement in one model does not establish a universal internal-mRNA writer, and reader-defined mechanisms need not resolve the enzyme depositing every site. Mapped sites, writer-associated enrichment, and directly demonstrated catalytic deposition therefore represent distinct evidence levels.

### 3.4 Modified circRNAs and tRNA fragments

M^7^G can influence cancer biology through RNAs generated from or interacting with canonical substrates. In prostate cancer, METTL1 depletion reduces tRNA m^7^G and increases a class of 5′tRNA-derived small RNAs that redirects translation toward tumor-suppressive and immune programs, increases pro-inflammatory immune infiltration, and improves immunotherapy responses in preclinical models (García-Vílchez et al., 2023a). In bladder cancer, an m^7^G-modified 3′tRNA fragment derived from LysTTT binds annexin A2, promotes its Tyr24 phosphorylation and nuclear localization, and enhances malignant behavior *in vitro* and *in vivo* (Ying et al., 2024). Thus, tRNA methylation can control both decoding and the production or activity of regulatory fragments.

Circular RNAs (circRNAs) constitute a second noncanonical layer. METTL1-mediated m^7^G protects circKDM1A from degradation, and mutation of its m^7^G site weakens AKT-pathway activation and colorectal cancer growth, invasion, and migration (Sun et al., 2024). Another colorectal cancer study reported that circ_0003323 binds METTL1, increases cellular m^7^G and KLK6 expression, and cooperates with METTL1 to promote tumor growth (Zhang M. et al., 2026). These observations expand the substrate network, although circRNA-specific stoichiometry and direct site validation will be needed before this mechanism can be generalized.

The noncoding network also includes modified long non-coding and microRNAs. WDR4-associated m^7^G was reported to stabilize ZFAS1 in laryngeal cancer, while ZFAS1 increases RBFOX2 and RBFOX2 reinforces WDR4 expression, creating a proposed modification-linked positive-feedback circuit (Lu et al., 2025). In HCC, METTL1 stabilizes m^7^G-enriched circIPP2A2, which scaffolds Hornerin and PI3K to activate AKT-GSK3β signaling (Guo et al., 2024). In bladder cancer, METTL1 promotes m^7^G-dependent processing of miR-760, which lowers the tumor suppressor ATF3 and supports tumor growth and metastasis (Xie et al., 2022). These pathways differ from tRNA decoding because the regulatory product may be a fragment, scaffold, or processed small RNA. A modified parental tRNA does not prove that every derived fragment retains the mark, and circRNA binding to METTL1 does not itself identify a methylated substrate.

### 3.5 Cap methylation and rRNA-associated pathways

The RNMT-dependent cap pathway can generate genotype-selective dependencies. Partial RNMT depletion has little effect on non-transformed mammary epithelial cells but reduces proliferation and increases apoptosis in breast cancer cells carrying oncogenic PIK3CA mutations; expression or inhibition experiments indicate that PI3Kα signaling determines this dependence (Dunn et al., 2019). These findings suggest that a basic RNA-processing step may acquire a therapeutic window when oncogenic signaling raises the demand for capped transcripts.

MYC can increase cap methylation beyond its transcriptional effects by recruiting RNMT to promoters of Wnt-β-catenin pathway genes, thereby increasing their translational capacity (Posternak et al., 2017). A B7-H6-c-MYC circuit in glioma stem-like cells also increases RNMT expression and supports proliferation, although that study did not directly quantify transcript-specific cap methylation (Chen et al., 2020). Cap readers extend this axis: NCBP2 enhances c-JUN translation and MEK-ERK signaling in pancreatic ductal adenocarcinoma, with genetic manipulation validating its effect on tumor growth (Xie et al., 2023). Pan-cancer profiling and functional depletion further identify the cap-binding protein CBP20 as a cell-cycle dependency and nominate compounds that phenocopy its loss (Cho et al., 2025).

rRNA-associated m^7^G remains less well defined in cancer. METTL1 and BUD23 are overexpressed in advanced clear-cell renal cell carcinoma, and depletion of either protein suppresses proliferation, colony formation, and migration; the study also detected METTL1-associated m^7^G on mRNAs encoding tumor suppressors (Su et al., 2025). Because BUD23 functions in ribosome biogenesis and METTL1 has multiple proposed RNA substrates, catalytic rescue and site-specific analyses are required to partition rRNA, tRNA, and mRNA contributions.

WBSCR22 underscores why rRNA-associated proteins warrant similar caution. WBSCR22 activates AKT-GSK3β-β-catenin signaling downstream of miR-146b-5p in glioma, but direct dependence on its rRNA methyltransferase activity was not demonstrated (Chi et al., 2020). Conversely, WBSCR22 and TRMT112 suppress pancreatic cancer growth and invasion through transcriptional repression of ISG15, revealing a tumor-restrictive function that may also be separable from rRNA m^7^G catalysis (Khan et al., 2022). Cap recognition has a defined biochemical substrate, whereas cancer phenotypes assigned to rRNA-associated proteins often lack catalytic partitioning. Opposite WBSCR22 phenotypes therefore cannot be reconciled solely by invoking higher or lower rRNA m^7^G.

### 3.6 Substrate-dependent tumor restriction

The prevailing oncogenic model is not universal across cancer types. In breast cancer, METTL1 and WDR4 were reported to be reduced, and restoration of catalytic METTL1 increased m^7^G on 19 tRNAs, favored codon-dependent translation of GADD45A and RB1, blocked cell-cycle progression, and enhanced the antitumor effect of the CDK4/6 inhibitor abemaciclib (Du et al., 2024). This finding contrasts with the growth-promoting tRNA programs observed in several other tumors.

Other RNA compartments also show directionally opposite effects. A lung-cancer study associated TRMT10C with increased circFAM126A m^7^G enrichment and RNA stability; however, direct catalytic writer activity of TRMT10C remains to be established. The circRNA suppresses lung cancer glycolysis and growth by promoting HSP90 ubiquitination and reducing AKT1 expression (Zhao et al., 2024). The cap-binding paralogue eIF4E3 recognizes the m^7^G cap through an atypical structural mode and competes with oncogenic eIF4E, producing tumor-suppressive effects that are lost in some high-eIF4E cancers (Osborne et al., 2013).

This discrepancy may reflect tissue-specific tRNA pools, codon usage, baseline writer abundance, or the identity of transcripts positioned to benefit from altered decoding. The opposing phenotypes are compatible with a nonlinear response, but they do not demonstrate a dose-response relationship within the same experimental system. m^7^G status should therefore be interpreted through substrate-level and proteomic consequences rather than writer expression alone. Substrate-level analysis requires identification of the modified RNA, catalytic dependence, the relevant effector, and the favored protein program. Lung cancer models link codon-biased translation to oncogenic outputs, whereas breast cancer models link the same writer complex to GADD45A/RB1-dependent cell-cycle restraint. A nonlinear response can be distinguished from substrate selection only by graded, matched perturbations that measure site occupancy, tRNA abundance, translation, and phenotype in the same model. Writer expression alone cannot support a pan-cancer ranking of m^7^G activity.

## 4 m^7^G-dependent tumor adaptation and therapeutic resistance

RNA-specific m^7^G mechanisms converge on a limited set of malignant capabilities. Growth and dissemination depend on selective translation or stabilization of cell-cycle, signaling, adhesion, and metabolic transcripts, whereas survival under stress requires autophagy, DNA repair, and preservation of the tRNA pool. These cell-intrinsic programs also alter cytokine output and metabolic communication with immune and stromal cells (Figure 2; Table 2). Under treatment, the same substrate-dependent programs can sustain residual cells or alter sensitivity through repair, signaling, and immune adaptation (Figure 3; Table 3).

**FIGURE 3:**
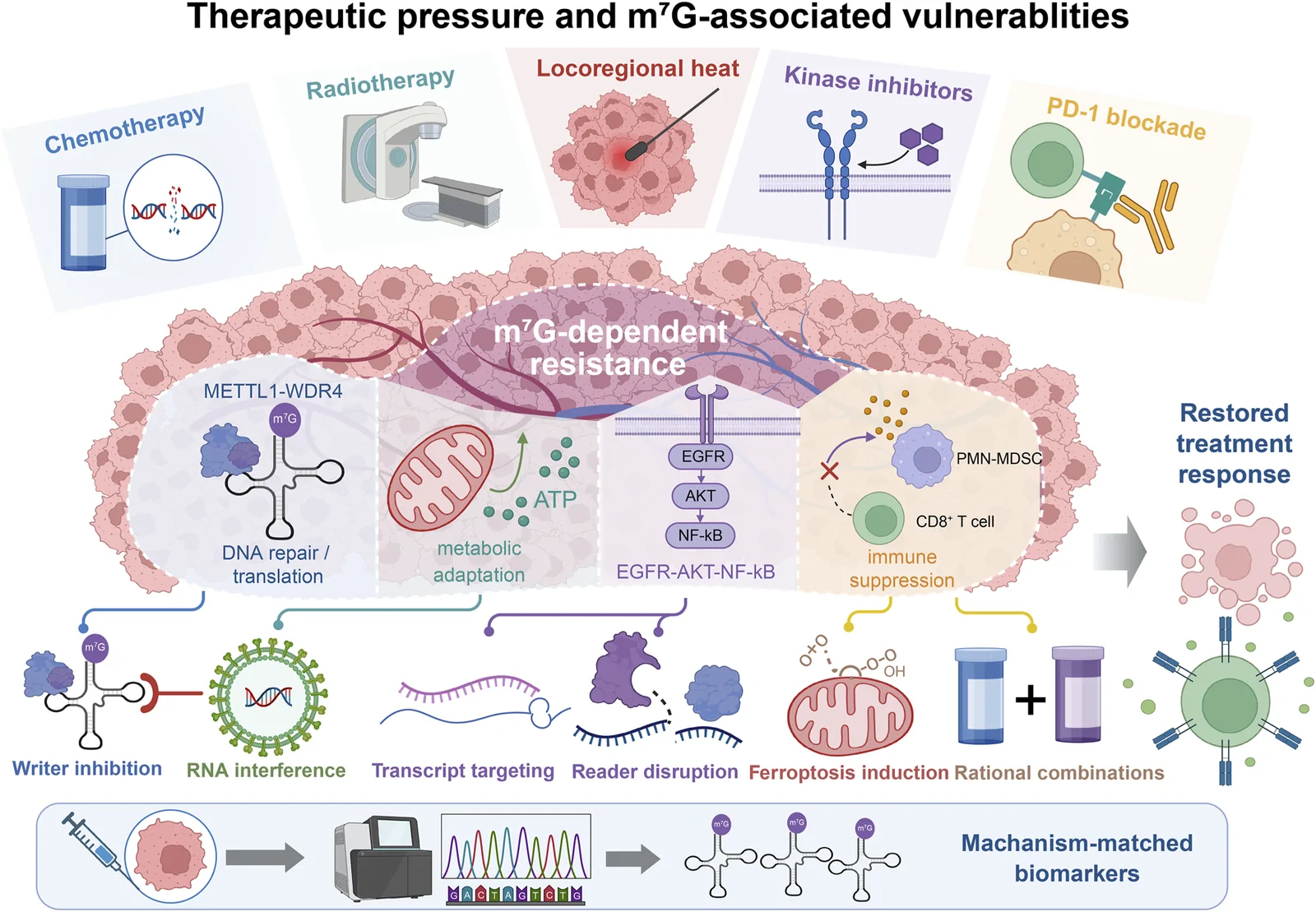
Therapeutic pressure and m^7^G-associated vulnerabilities. Chemotherapy, radiotherapy, locoregional heat, kinase inhibition, and immune checkpoint blockade act as upstream pressures that select translational adaptation, DNA damage and repair, metabolic and signaling adaptation, immune adaptation, and stress adaptation through m^7^G-dependent survival programs. Potential interventions include catalytic writer inhibition, RNA interference, transcript-directed targeting, reader disruption, ferroptosis induction, and mechanism-guided combination therapy. Created in BioRender.

**TABLE 3:** m^7^G-associated treatment response, resistance, and therapeutic intervention. The adaptive-mechanism column uses five categories: translational adaptation, DNA damage and repair, metabolic and signaling adaptation, immune adaptation, and stress adaptation. The causal-status column separately records whether the reported effect is directly m^7^G-dependent, regulator-associated, cap/reader-directed, or m^7^G-independent.

Adaptive mechanism	Causal status	Cancer type	Therapy or intervention	Resistance mechanism or target	Effect on response	Evidence basis	Ref.
Stress adaptation	Direct m^7^G-dependent	Cervical cancer cells	5-Fluorouracil	NSUN2-METTL1-dependent tRNA protection	Dual depletion increases drug sensitivity	HeLa cells	(Okamoto et al., 2014)
Stress adaptation	m^7^G-associated	Colon cancer	Cisplatin	METTL1-miR-149-3p-S100A4-p53 regulation	METTL1 restoration increases sensitivity	Resistant cell models	(Liu et al., 2019)
Stress adaptation	m^7^G-associated	NSCLC	SN-38; 5-fluorouracil	WBSCR22-dependent drug response	WBSCR22 loss reduces sensitivity	Cell lines; xenografts	(Yan et al., 2015)
Translational adaptation	Direct m^7^G-dependent	Nasopharyngeal carcinoma	Cisplatin; docetaxel	tRNA m^7^G-dependent WNT-β-catenin translation	METTL1-WDR4 promotes chemoresistance	Clinical samples; cells; mice	(Chen et al., 2022a)
Translational adaptation	Direct m^7^G-dependent	Osteosarcoma	Doxorubicin	Codon-biased translation of ECM-remodeling proteins	Writer depletion restores drug response	Clinical samples; cells; mice	(Wang et al., 2023b)
Translational adaptation	Direct cap mechanism	CRC	5-Fluorouracil	eIF4E-dependent integrin β1 translation	Cap-reader signaling promotes resistance	Clinical samples; cell models	(Niu et al., 2018)
Immune adaptation	m^7^G-associated	Oxaliplatin-resistant CRC	Natural killer cells	miR-146b-5p-mediated WBSCR22 repression	Natural killer cells suppress resistant tumors	Co-culture; mouse xenografts	(Zhao et al., 2018)
Immune adaptation	Direct m^7^G-dependent	ICC	Anti-PD-1	METTL1-dependent CXCL8/Cxcl5 translation and MDSCs	METTL1 blockade improves checkpoint efficacy	Clinical samples; genetic mice	(Liu et al., 2023)
Immune adaptation	m^7^G-associated	Melanoma	Anti-PD-1	POU4F1-METTL1-PKM2 glycolysis	Glycolytic m^7^G signaling drives primary resistance	Patient samples; cells; mice	(Liu et al., 2025c)
Immune adaptation	Direct m^7^G-dependent	Post-ablation HCC	TGF-β or MDSC blockade	METTL1-TGF-β2-mediated immune suppression	Pathway blockade prevents recurrence	Clinical samples; cells; mice	(Zeng et al., 2023)
Metabolic and signaling adaptation	m^7^G-associated	Osteosarcoma	Doxorubicin	ALKBH1-FZD8-Wnt-β-catenin regulation	ALKBH1 increases chemosensitivity	Cells; mouse models	(Li et al., 2025c)
DNA damage and repair	Direct m^7^G-dependent	HCC	Radiotherapy	tRNA m^7^G-dependent DNA-PKcs and LIG4 translation	METTL1 enhances NHEJ and radioresistance	Clinical samples; cells; mice	(Liao et al., 2023)
Translational adaptation	Direct m^7^G-dependent	HCC	Radiofrequency ablation	tRNA m^7^G-dependent SLUG and SNAIL translation	METTL1 drives post-ablation metastasis	Clinical samples; organoids; PDX; mice	(Zhu et al., 2023b)
Metabolic and signaling adaptation	m^7^G-associated	Esophageal cancer	Radiotherapy	PFKFB3 mRNA stabilization and glycolysis	METTL1 promotes radioresistance	Cells; mouse xenografts	(Xiao and Hou and Wang and Huang and Liu, 2025a)
Metabolic and signaling adaptation	Direct m^7^G-dependent	OSCC	Anlotinib	tRNA m^7^G-driven oxidative phosphorylation	METTL1 promotes acquired resistance	Resistant cells; transgenic mice	(Chen et al., 2024b)
Translational adaptation	Direct m^7^G-dependent	HCC	Lenvatinib	tRNA m^7^G-dependent EGFR-pathway translation	METTL1 depletion restores sensitivity	Resistant cells; multiple mouse models	(Huang et al., 2023)
Metabolic and signaling adaptation	m^7^G-associated	NSCLC	Gefitinib	SCLT1 mRNA stabilization; NF-κB activation	METTL1-WDR4 promotes resistance	Clinical samples; cells; mice	(Zhou et al., 2025)
Metabolic and signaling adaptation	m^7^G-associated	Lung adenocarcinoma	Gefitinib	FOXM1 mRNA stabilization; PTPN13 repression	METTL1 reduces gefitinib sensitivity	Patient data; cells; xenografts	(Peng and Fu and Zhou and Duan, 2024)
Metabolic and signaling adaptation	m^7^G-associated	NSCLC	Osimertinib	CAF-HMGB1-METTL1-NET1 signaling	METTL1 depletion reverses stromal resistance	CAF co-culture; cells; mice	(Qian et al., 2026)
Stress adaptation	m^7^G-associated	HCC	Lenvatinib	WDR4-dependent TRIM28 and stemness	WDR4 promotes acquired resistance	Clinical samples; cells; mice	(Han et al., 2023)
Metabolic and signaling adaptation	m^7^G-independent	EGFR-TKI-resistant NSCLC	Competitive WDR4 peptide	PTPN23 ubiquitination; impaired receptor degradation	Peptide restores EGFR and c-MET degradation	Cells; metastatic mouse models	(Singh et al., 2023)
Stress adaptation	m^7^G-associated	Neuroblastoma	Dauricine	METTL1-SLC3A2 m^7^G and ferroptosis	Induces ferroptosis and suppresses growth	Cells; xenografts	(Tang and Huang and Li and Ren, 2026b)
DNA damage and repair	Direct m^7^G-dependent	Osteosarcoma	siMETTL1-doxorubicin-belinostat nano-drug	tRNA m^7^G and histone acetylation	Increases DNA damage and chemosensitivity	Cells; mouse tumors	(Qi et al., 2026)
Translational adaptation	Direct cap mechanism	eIF4E-dependent cancers	Ribavirin	Competitive mimicry of the m^7^G cap	Suppresses oncogenic translation and growth	Cells; mouse tumors; AML samples	(Kentsis and Topisirovic and Culjkovic and Shao and Borden, 2004)
Translational adaptation	Direct cap mechanism	Lung epithelial cancer model	4Ei-1	Disruption of eIF4E-cap binding	Blocks SNAIL1 translation and EMT	Cell-based EMT model	(Smith et al., 2015)
Translational adaptation	Reader-protein interaction	TP53-wild-type cancer models	Compound 094 plus cytotoxic therapy	Disruption of eIF4E-RBM38 binding	Restores TP53 translation and combination activity	Cells; tumor spheroids	(Lucchesi and Zhang and Gao and Shaw and Chen, 2023)
DNA damage and repair	m^7^G-associated	High-grade serous ovarian cancer	Microbial metabolite FAICAR	METTL1-BRCA1 m^7^G regulation	Reduces BRCA1 expression and tumor growth	Microbiome data; cells; xenografts	(Chen and He, 2025)

### 4.1 Malignant growth and metastatic plasticity

Growth-factor signaling is a recurring endpoint of m^7^G dysregulation. In bladder cancer, METTL1-mediated tRNA modification promotes translation of EGFR and EFEMP1, and re-expression of either target partially rescues the proliferation, migration, and invasion defects caused by METTL1 loss (Ying et al., 2021). Other models connect m^7^G to cyclins, PI3K/AKT, WNT, and mTOR, but the upstream RNA event differs between codon-biased translation and internal mRNA stabilization. The shared phenotype therefore arises through several RNA routes rather than one conserved target transcript.

Prostate cancer cells likewise show coordinated METTL1-WDR4 elevation, increased tRNA m^7^G, PI3K/AKT/mTOR activity, and expression of MYC and VEGF, although the functional evidence was derived mainly from one cell-line model (Deng W. et al., 2025). In glioma, WDR4 depletion causes G1 arrest and reduces PI3K/AKT-linked CDK1 and CDK2 expression in cells and xenografts, but catalytic m^7^G dependence remains unresolved (Liu et al., 2025b). Colorectal cancer data provide a more focused cell-cycle connection in which METTL1 attenuates CHEK2-induced G1-S arrest (Jiang et al., 2024). These observations link RNA regulation to cell-cycle progression through different downstream regulators. Regulator-associated signaling remains mechanistically unresolved without a defined modified substrate.

Metastatic potential can be reinforced by positive feedback between m^7^G machinery and oncogenic signaling. In gastric cancer, YY1 induces METTL1 and WDR4, while METTL1-WDR4-dependent m^7^G stabilizes PIK3C2B and AKT transcripts and feeds back to increase YY1, supporting migration and proliferation in cell and animal models (Wang J. et al., 2025). In metastatic colorectal cancer, METTL1 stabilizes ICAM1 mRNA through m^7^G and thereby enhances invasive behavior (Cai et al., 2025). Proteomic, single-cell, tRNA-mapping, and nascent-chain analyses further show that METTL1 is highest in colorectal liver metastases and promotes translation of CCND3 and PI3K/AKT-related transcripts in a catalytic-activity-dependent manner (Chen et al., 2026).

WDR4 can also promote dissemination through mechanisms that are not yet tied to methyltransferase activity. In colorectal cancer, WDR4 binds GSK3β and activates β-catenin signaling in cell and xenograft models (Wang H. et al., 2025). In bladder cancer, WDR4 scaffolds DDX20 and EGR1 to repress ARRB2 transcription, thereby increasing lymphatic metastasis and independently predicting nodal involvement (Wang G. et al., 2023). At the m^7^G-cap reader level, phosphorylation of eIF4E promotes translation of SNAIL and MMP3 and is required for epithelial-mesenchymal transition and lung metastasis in a mammary tumor model (Robichaud et al., 2015). Dissemination thus converges on adhesion and signaling outputs while retaining a mixture of RNA-dependent and protein-scaffolding mechanisms. A metastatic phenotype following WDR4 depletion is insufficient to identify which route is responsible.

M^7^G does not drive proliferation and metastasis through a universal downstream oncogene. Different RNA substrates instead converge functionally on cell-cycle progression, growth-factor signaling, adhesion, and epithelial plasticity. Coupling motility to the biosynthetic demands of colonization may help disseminating cells maintain growth and survival.

### 4.2 Metabolic and stress adaptation

M^7^G-dependent RNA regulation can reshape carbon and nitrogen metabolism. In gastric cancer, METTL1-WDR4 maintains modified tRNAs that favor translation of oxidative-phosphorylation genes, including SDHAF4, thereby increasing respiratory complex II activity and tumor progression (Xu X. et al., 2025). In glioma, internal m^7^G deposition on PGK1 mRNA increases transcript stability, glycolysis, proliferation, and xenograft growth (Fan et al., 2025). In HCC, METTL1 stabilizes ASNS mRNA, increases asparagine synthesis, activates mTOR signaling, and promotes malignant progression (Xing et al., 2026).

The stroma can also impose metabolic effects. Cancer-associated fibroblast-derived exosomes increase METTL1 in colorectal cancer cells, where m^7^G-dependent stabilization of the glutamine transporter SLC1A5 promotes glutamine consumption, stem-like behavior, tumor growth, and lung metastasis (Zhang S. et al., 2026). These mechanisms connect m^7^G to oxidative phosphorylation, glycolysis, and amino-acid acquisition, suggesting that substrate-specific RNA regulation helps tumors adapt metabolism to local nutrient availability.

In cutaneous squamous cell carcinoma, METTL1-dependent m^7^G stabilizes ATF4 mRNA, and ATF4 rescue restores glycolysis and malignant phenotypes after METTL1 depletion (Zhang et al., 2025a). These findings add an integrated stress-response transcription factor to the metabolic m^7^G network and further illustrate that glycolytic output can be regulated through direct mRNA stability as well as tRNA-driven translation. The convergence is metabolic flexibility, not a uniform preference for glycolysis. tRNA-dependent respiratory translation and reported internal-mRNA stabilization of glycolytic or amino-acid programs indicate that the favored substrate helps determine the metabolic direction. This flexibility connects nutrient use to the capacity to survive stress.

Maintenance of translation under deteriorating growth conditions requires an intact tRNA pool and adaptation of cellular recycling. The METTL1-WDR4 axis supports both processes in the reported cancer models. In esophageal squamous cell carcinoma, reduced tRNA m^7^G suppresses translation of RPTOR/ULK1/autophagy-pathway transcripts, whereas genetically engineered mouse models confirm that catalytic METTL1 promotes tumorigenesis (Han et al., 2022). More broadly, tRNA m^7^G protects tRNAs from stress-induced cleavage into 5′fragments, and loss of METTL1 activates stress responses, increases cytotoxic stress, and restricts tumor growth (García-Vílchez et al., 2023b). Modification of the tRNA pool therefore preserves both decoding capacity and resistance to RNA fragmentation.

Internal mRNA regulation can yield a distinct autophagy phenotype. In colorectal cancer, a P300-ETV4 transcriptional complex raises WDR4 expression, WDR4-associated m^7^G was reported to stabilize SPP1 mRNA, and the resulting pathway suppresses autophagy while promoting growth and metastasis (Deng X. et al., 2025). Whether autophagy is maintained or suppressed thus depends on the relevant RNA substrate and tumor state. The opposing autophagy outputs suggest that growth support and stress buffering can involve different downstream programs; autophagy activation alone is insufficient to predict the effect of writer inhibition.

Ferroptotic stress may represent another downstream outcome. GEMIN5 binds SHMT2 mRNA in colorectal cancer cells, increases an m^7^G-associated signal and transcript stability, and suppresses ferroptosis through SHMT2 and Wnt-β-catenin signaling, although the proposed writer activity of a cap-binding protein requires further biochemical clarification (Wang Z. et al., 2025). The broader relevance of ferroptotic control to tumor adaptation is also supported by evidence from epithelial ovarian cancer, where ACSL4 enhanced ferroptosis and promoted M1 macrophage polarization, linking tumor-cell susceptibility to lipid peroxidation with changes in the immune microenvironment (Qi et al., 2025). These data implicate ferroptotic tolerance without establishing GEMIN5 as an m^7^G methyltransferase. Metabolic and RNA-stress programs therefore provide several routes to survival, whose importance may change when treatment imposes additional damage.

### 4.3 DNA damage response and genome maintenance

DNA repair provides a direct link between RNA modification and survival after genotoxic treatment. In HCC, METTL1-dependent tRNA m^7^G preferentially increases translation of DNA-dependent protein kinase catalytic subunit (DNA-PKcs) and DNA ligase IV after irradiation, strengthens non-homologous end joining (NHEJ), and supports radioresistance; high tumor METTL1 expression correlates with poor outcome among radiotherapy-treated patients (Liao et al., 2023). The codon composition of the repair transcripts connects this response to tRNA decoding. Thus, m^7^G acts through the supply of repair proteins rather than modification of DNA itself, enabling the RNA pathway to influence recovery from radiation-induced double-strand breaks.

Repair-protein translation is also therapeutically relevant in osteosarcoma. A carrier-free nano-epidrug combines METTL1 silencing with doxorubicin and belinostat to suppress tRNA-dependent translation of DNA-repair proteins while increasing treatment-induced DNA damage (Qi et al., 2026). The combination supports repair-associated chemosensitization, but its activity integrates RNA regulation, chromatin effects, and drug delivery; it cannot be attributed entirely to METTL1 inhibition.

Evidence involving BRCA1 is less direct. In high-grade serous ovarian cancer, METTL1 depletion reduces BRCA1 expression, and BRCA1 re-expression reverses growth suppression in an m^7^G-associated pathway (Chen and He, 2025). These findings nominate a repair-related transcript but do not establish altered homologous recombination capacity or resistance to platinum or poly (ADP-ribose) polymerase inhibitors.

These findings suggest that repair-pathway activity may help identify tumors in which RNA-directed intervention complements genotoxic treatment. NHEJ dependence and BRCA1 expression should not be treated as interchangeable biomarkers. The combination results support a preclinical treatment hypothesis whose clinical value will depend on tumor-specific repair requirements and the ability to preserve repair in normal tissues.

### 4.4 Therapeutic resistance and treatment-induced adaptation

Therapy places acute stress on RNA metabolism, protein synthesis, DNA repair, and cell-state plasticity. m^7^G-dependent circuits can either buffer this stress or shift RNA output toward treatment-specific survival proteins. Constitutive tumor dependence and adaptation induced by therapy are distinct; their relevance to resistance depends on the substrate and protein program required during that treatment.

Chemotherapy resistance can emerge when codon-biased translation favors survival and cellular plasticity. In nasopharyngeal carcinoma, ARNT-induced METTL1-WDR4 increases tRNA m^7^G, improves decoding of m^7^G-tRNA-associated codons, activates WNT/β-catenin signaling, and drives epithelial-mesenchymal transition and resistance to cisplatin and docetaxel (Chen B. et al., 2022). In osteosarcoma, METTL1-WDR4 favors translation of extracellular-matrix remodeling proteins and promotes doxorubicin resistance, supporting combination strategies that inhibit the writer while preserving cytotoxic exposure (Wang Z. et al., 2023). These examples link tRNA decoding to resistance while retaining distinct downstream programs.

Cap- and rRNA-associated proteins also influence chemotherapy response. eIF4E-dependent translation of integrin β1 promotes 5-fluorouracil resistance in colorectal cancer cells and correlates with poor outcome in patient tissues (Niu et al., 2018). Natural killer cells suppress oxaliplatin-resistant colorectal tumors by increasing miR-146b-5p and lowering WBSCR22, whereas WBSCR22 depletion paradoxically reduces sensitivity to SN-38 and 5-fluorouracil in H460 lung cancer cells (Yan et al., 2015; Zhao et al., 2018). A recent osteosarcoma study further proposes that ALKBH1 increases doxorubicin sensitivity through an m^7^G-associated FZD8-Wnt-β-catenin pathway, but direct demethylation chemistry at the nominated site remains to be established (Li X. et al., 2025). tRNA preservation and regulator-associated signaling also influence drug sensitivity. Combined depletion of NSUN2 and METTL1 sensitizes HeLa cells to 5-fluorouracil, whereas dephosphorylated forms of the enzymes that preserve tRNA-modifying activity reduce drug sensitivity (Okamoto et al., 2014). A different direction was observed in cisplatin-resistant colon cancer cells, where METTL1 is reduced and its overexpression restores cisplatin sensitivity through a miR-149-3p-S100A4-p53 axis (Liu et al., 2019). Because the latter study did not establish a site-resolved tRNA or mRNA m^7^G mechanism, it should be interpreted as METTL1-associated rather than definitive m^7^G causality.

Reader-mediated RNA partitioning can also reverse the expected direction of stress adaptation. QKI7 recruits internal m^7^G-modified transcripts to G3BP1-dependent stress granules and reduces translation of Hippo-pathway genes, increasing doxorubicin sensitivity (Zhao et al., 2023). Stress-granule formation therefore does not invariably protect cancer cells; its effect depends on which transcripts are sequestered. Alongside tRNA preservation, this reader-controlled route explains why the same RNA modification can participate in resistance or sensitization through distinct substrates and effectors.

Metabolic adaptation can sustain radioresistance. Esophageal cancer cells use a metabolism-centered route in which METTL1-dependent m^7^G stabilizes PFKFB3 mRNA, increases glycolytic flux and stem-like behavior, and reduces the response to irradiation *in vitro* and in xenografts (Xiao C. et al., 2025). Incomplete local treatment can induce metastatic plasticity: insufficient radiofrequency ablation increases METTL1-WDR4 and tRNA m^7^G in patient-derived xenografts, organoids, clinical tissues, and cell models, promoting codon-dependent translation of SLUG and SNAIL and post-ablation metastasis (Zhu S. et al., 2023). This local treatment stress can also remodel antitumor immunity. After insufficient radiofrequency ablation, heat-induced METTL1 increases translation of transforming growth factor-β2, expands polymorphonuclear myeloid-derived suppressor cells, excludes CD8^+^ T cells, and accelerates HCC recurrence; blockade at several points in this axis restores antitumor immunity in mice (Zeng et al., 2023).

Kinase inhibitors reveal both tRNA- and mRNA-centered resistance mechanisms. Lenvatinib-resistant HCC cells upregulate METTL1-WDR4, and catalytic tRNA m^7^G promotes translation of EGFR-pathway genes; METTL1 depletion restores apoptosis and drug sensitivity across several mouse models (Huang et al., 2023). More broadly, pharmacological studies in HCC have shown that kinase-inhibitor resistance can also be coupled to cell-death plasticity, as AZ-628 resensitized donafenib-resistant HCC cells while modulating tyrosine kinase signaling, epithelial–mesenchymal transition, apoptosis, and ferroptosis (Yu et al., 2025). In non-small cell lung cancer (NSCLC), METTL1-WDR4-dependent internal m^7^G stabilizes SCLT1 mRNA, activates nuclear factor-κB (NF-κB) signaling, and promotes gefitinib resistance (Zhou et al., 2025). Cancer-associated fibroblasts can also secrete high-mobility group box 1 to increase METTL1 in NSCLC cells, where NET1 m^7^G activates AKT/NF-κB signaling and confers resistance to osimertinib (Qian et al., 2026). Accordingly, targeted-therapy resistance can arise within the tumor cell or be instructed by the microenvironment.

Additional resistance circuits extend this pattern. In oral squamous cell carcinoma, METTL1-dependent tRNA m^7^G drives a switch from glycolysis to oxidative phosphorylation and confers acquired resistance to anlotinib in cell and transgenic mouse models (Chen J. et al., 2024). In lung adenocarcinoma, METTL1 stabilizes FOXM1 mRNA, FOXM1 represses PTPN13, and the resulting axis reduces gefitinib sensitivity (Peng et al., 2024). In HCC, WDR4 increases TRIM28 expression and stem-like properties during lenvatinib resistance, although the specific modified RNA was not resolved (Han et al., 2023). WDR4 can also promote EGFR-tyrosine kinase inhibitor resistance independently of m^7^G by ubiquitinating PTPN23 and preventing lysosomal degradation of EGFR and c-MET; a competitive peptide restores receptor degradation in resistant NSCLC models (Singh et al., 2023). These examples separate receptor-pathway translation and RNA stability from protein turnover. The m^7^G-independent WDR4–PTPN23 pathway provides a bypass mechanism without establishing resistance through RNA methylation.

Tumor-cell-intrinsic m^7^G-associated pathways can alter immune recognition via metabolic and transcriptional feedback. In colorectal cancer, METTL1-dependent m^7^G of PKM mRNA increases PKM2, glycolysis, histone H3 lysine nine lactylation, and CD155 expression, producing a positive feedback loop associated with immune escape (Wang et al., 2024). In breast cancer, a multi-RNA-modification score identified WDR4 as a major contributor to an immunosuppressive microenvironment and resistance to chemotherapy and programmed death-ligand one blockade; functional WDR4 inhibition reduced tumor growth and mTORC1 signaling (Luo et al., 2025). Not every immune function of an m^7^G-associated protein is modification dependent. In HCC-associated macrophages, cytoplasmic WDR4 interacts with eIF4E2 to enhance ABCA1 translation and cholesterol efflux independently of m^7^G, maintaining a protumor macrophage state and limiting programmed cell death protein 1 (PD-1) blockade (Liu et al., 2025a). This immune-cell-intrinsic protein interaction differs from the tumor-cell RNA circuits that alter metabolites or surface ligands. The cellular source and catalytic requirement therefore determine whether an immune phenotype can be assigned to m^7^G itself.

M^7^G can shape immunotherapy responses by regulating chemokines and metabolic exclusion. In ICC, tumor-cell METTL1 selectively increases translation of CXCL8 in humans and Cxcl5 in mice, recruits polymorphonuclear myeloid-derived suppressor cells, and limits PD-1 blockade; genetic or chemical interference with this axis improves anti-PD-1 efficacy in multiple preclinical models (Liu et al., 2023). In melanoma, POU4F1 increases METTL1 and m^7^G on PKM2 mRNA, enhances glycolysis, reduces cytotoxic immune-cell infiltration, and promotes primary anti-PD-1 resistance in patient samples and experimental models (Liu L. et al., 2025).

Across these treatments, resistance and sensitization arise from RNA programs that also regulate malignant growth, stress tolerance, and the tumor microenvironment. The direction of response depends on the favored RNA and protein program, so elevated writer activity cannot by itself predict resistance. Therapeutic selection should therefore follow the demonstrated RNA substrate, protein output, and cellular compartment rather than METTL1 or WDR4 abundance alone.

## 5 Biomarkers and therapeutic targeting

### 5.1 Biomarker development

Most biomarker studies have measured METTL1 or WDR4 expression rather than the modification itself (Table 4). In two HCC cohorts totaling 892 patients, high METTL1 expression was associated with larger tumors, vascular invasion, increased alpha-fetoprotein, reduced PTEN signaling, and unfavorable survival (Tian et al., 2019). In cutaneous melanoma, public datasets, single-cell analyses, and cell experiments link METTL1 amplification and MYC regulation to aggressive behavior, low CD8^+^ T Cell infiltration, poor prognosis, and reduced immunotherapy response (Xia and Yin, 2025). A prostate cancer model based on METTL1 and WDR4 expression also correlated with survival and immune infiltration, although its evidence remains primarily computational with limited *in vitro* validation (Kong et al., 2025).

**TABLE 4:** Clinical and biomarker evidence for m^7^G-related regulators in cancer. Classification distinguishes writer/cofactor expression, cap-reader abundance, and germline susceptibility. The validation column summarizes the type and level of functional evidence.

Classification	Biomarker	Cancer type	Sample or design	Clinical value	Validation or limitation	Ref.
Writer/cofactor expression	METTL1 expression	HCC	Two retrospective cohorts; 892 patients	Independent adverse prognostic factor	Tissue-level expression association	(Tian et al., 2019)
Writer/cofactor expression	METTL1 expression	Cutaneous melanoma	Public cohorts; single-cell data; cell validation	Association with prognosis and immunotherapy response	Retrospective; functional assays support oncogenicity	(Xia and Yin, 2025)
Writer/cofactor expression	METTL1-WDR4 expression	Prostate cancer	TCGA cohorts; *in vitro* validation	Two-gene survival model	Predominantly computational; limited external validation	(Kong et al., 2025)
Writer/cofactor expression	METTL1 expression	Clear-cell renal cancer	TCGA modeling; immunohistochemistry	Association with diagnostic and prognostic features	Expression-based clinical association	(Liu et al., 2025d)
Writer/cofactor expression	METTL1 expression	Glioma	Five databases; tissues; U87 cells	Grade and survival association	Single-cell-line functional validation	(Li et al., 2021)
Writer/cofactor expression	RNMT expression	Pan-cancer; lung SCC	Multi-omics; machine learning; qPCR and immunoblotting	Association with prognosis and immune features	Expression-level assessment of cap-methyltransferase abundance; functional cap-methylation activity was not directly measured	(Huang et al., 2024)
Cap-reader abundance	eIF4E protein	Multiple cancers	Multi-tumor tissue microarray	Correlates with VEGF and cyclin D1	Cross-sectional protein association	(Yang and Hewitt and Steinberg and Liewehr and Swain, 2007)
Germline susceptibility	WDR4 rs6586250	Wilms tumor	Five-center case-control study	Increased susceptibility in Chinese children	414 cases; population replication required	(Deng et al., 2023)
Germline susceptibility	WDR4 rs15736	Pediatric glioma	Three-center case-control study	Reduced susceptibility in selected subgroups	314 cases; ethnicity-specific evidence	(Zhu et al., 2023a)

Independent biomarker studies extend this pattern but differ in evidentiary depth. METTL1 expression has been associated with clear-cell renal cancer progression using machine learning and immunohistochemical validation, and with glioma grade and outcome using public cohorts, tissue staining, and limited cell assays (Li et al., 2021; Liu Y. et al., 2025). RNMT expression correlates with prognosis and immune features across several cancers, with transcript and protein upregulation validated in lung squamous cell carcinoma but without direct cap-methylation measurements (Huang et al., 2024). Earlier multi-tumor tissue microarrays linked eIF4E abundance with VEGF and cyclin D1 across several malignancies, supporting cap-reader activity as a broad translational biomarker rather than an m^7^G-site-specific assay (Yang et al., 2007).

Germline variation provides another biomarker layer. A five-center case-control study associated the WDR4 rs6586250 variant with Wilms tumor susceptibility, while a three-center pediatric glioma study associated WDR4 rs15736 with reduced risk and altered WDR4 expression (Deng et al., 2023; Zhu J. et al., 2023). These associations require population replication and do not establish that altered cancer risk is mediated by RNA m^7^G.

Expression-based biomarkers are practical but biologically nonspecific. Expression measures identify regulator abundance; functional activity measures require RNA-class-specific occupancy, catalytic output, or a validated translation signature. A clinically informative assay should specify whether it measures writer abundance, catalytic activity, global m^7^G, an RNA-class-specific signal, a modified site, or a downstream translation signature. Tissue composition is another source of ambiguity because tumor cells, fibroblasts, and immune cells can use m^7^G-associated proteins differently. Prospective validation should therefore pair spatially resolved protein expression with RNA-type-specific modification and treatment outcome.

### 5.2 Intervention strategies by target layer

#### 5.2.1 Targeting writer abundance or catalytic activity

Current interventions primarily target METTL1 indirectly or use RNA delivery rather than a selective catalytic inhibitor. Dauricine suppresses METTL1 expression in neuroblastoma, reduces m^7^G modification and expression of SLC3A2, increases ferroptosis, and limits xenograft growth, although molecular docking and expression changes do not yet demonstrate direct selective inhibition of METTL1 (Tang et al., 2026). Dauricine can affect several pathways that contribute to ferroptotic death, so these data do not establish selective METTL1 inhibition.

RNA-based combination therapy offers a more direct route. A carrier-free nano-epidrug combining METTL1 small interfering RNA, doxorubicin, belinostat, and a tumor-targeting lipid increases tumor accumulation, disrupts translation of DNA-repair proteins, opens chromatin through histone deacetylase inhibition, and enhances osteosarcoma chemosensitivity *in vivo* (Qi et al., 2026). This design demonstrates how m^7^G inhibition can be paired with an existing treatment and a second epigenetic perturbation, but its complexity also raises manufacturing, biodistribution, and attribution challenges.

METTL1-directed metabolic interventions are more recent. The microbial metabolite 5-formamidoimidazole-4-carboxamide ribotide was reported to reduce BRCA1 expression in an m^7^G-associated pathway and inhibit high-grade serous ovarian cancer in cells and xenografts; interaction with METTL1 was proposed on the basis of molecular docking, while selectivity and pharmacokinetic exposure remain undefined (Chen and He, 2025).

#### 5.2.2 Targeting cap recognition and reader-associated translation

Cap-reader interventions have a longer experimental history. Ribavirin mimics the m^7^G cap, competes for eIF4E binding, suppresses eIF4E-dependent oncogenic translation, and reduces tumor growth in mouse and patient-derived leukemia models (Kentsis et al., 2004). A chemical antagonist of the eIF4E-cap interaction blocks recruitment of SNAIL1 mRNA to polysomes and prevents transforming growth factor-β1-induced epithelial-mesenchymal transition (Smith et al., 2015). A distinct small molecule disrupts the eIF4E-RBM38 complex, restores wild-type TP53 translation, and cooperates with doxorubicin or 4EGI-1 to inhibit tumor spheroids (Lucchesi et al., 2023). Ribavirin and 4Ei-1 target cap recognition, whereas compound 094 acts on an eIF4E–protein interaction. These approaches do not directly inhibit m^7^G deposition and require different pharmacodynamic readouts from a METTL1 catalytic inhibitor.

#### 5.2.3 Transcript-directed strategies and prospective writer interventions

Potential strategies include structure-guided METTL1 inhibitors, disruption of METTL1-WDR4 assembly, selective degradation of the writer complex, antisense or CRISPR-based editing of an m^7^G-regulated transcript, and inhibition of a reader-effector interaction. Each approach carries a different safety profile. Blocking a tumor-restricted downstream RNA may offer greater selectivity than globally suppressing an enzyme required for normal tRNA stability. Transcript-directed intervention acts at the substrate or downstream effector layer and need not reduce global m^7^G. It remains a prospective strategy here rather than a clinically validated treatment.

### 5.3 Requirements for clinical translation

An actionable m^7^G target requires evidence beyond reduced tumor growth after writer knockdown. The intervention should show biochemical engagement, catalytic dependence, dose-response behavior, activity in patient-derived models, and a therapeutic window relative to normal proliferative and immune tissues. Resistance mechanisms should also be anticipated because tumors may compensate through alternative tRNAs, translation-initiation pathways, RNA readers, or metabolic rewiring. Reported interventions differ substantially in evidentiary support (Table 5). Genetic suppression, molecular docking, combination efficacy, and direct binding each support a different level of target validation.

**TABLE 5:** Intervention layer, demonstrated activity, and translational development priorities.

Intervention/ source	Target layer	Evidence supporting activity	Target engagement/ specificity context	Clinical-development priorities	References
Dauricine	Writer abundance, indirect	METTL1/SLC3A2 reduction, ferroptosis, xenograft inhibition	Docking and expression-based support for an indirect writer-related effect	Exposure-response, tumor selectivity, and matched normal-tissue effects	(Tang et al., 2026a)
siMETTL1–doxorubicin– belinostat nano-drug	Writer depletion plus combination	Tumor delivery, altered DNA-repair translation, chemosensitization in mice	RNA-interference-mediated writer depletion within a multi-payload formulation	Factorial payload controls, biodistribution, and combination therapeutic window	(Qi et al., 2026)
Ribavirin	Cap recognition	eIF4E interaction and antitumor activity in experimental models	Cap-recognition mechanism at eIF4E, distinct from writer-directed inhibition	Mechanism-linked exposure and selective oncogenic translation	(Kentsis et al., 2004)
4Ei-1	Cap recognition	SNAIL1 polysome recruitment and EMT suppression in a cell model	Cap-binding mechanism distinct from RNA writing	Patient-derived efficacy and systemic therapeutic window	(Smith et al., 2015)
Compound 094	Reader-protein interaction	eIF4E–RBM38 disruption and TP53 translation; tumor spheroids	eIF4E–RBM38 interaction targeting at the reader-associated translation layer	*In vivo* exposure, efficacy, and normal-tissue selectivity	(Lucchesi et al., 2023)
FAICAR	Proposed writer-associated intervention	Proposed METTL1 interaction; reduced BRCA1 expression; cell and xenograft activity	Docking prediction and expression effects; direct binding, enzyme-level inhibition, selectivity, and catalytic dependence remain unresolved	Pharmacokinetic exposure, dose-response target engagement, and safety	(Chen and He, 2025)
Competitive WDR4 peptide	m^7^G-independent protein pathway	Restores receptor degradation in resistant NSCLC models	WDR4–PTPN23 protein-regulatory mechanism, independent of RNA methylation	Delivery, exposure, and normal-tissue therapeutic window	(Singh et al., 2023)

Companion diagnostics should align with the mechanism of the drug. A METTL1 catalytic inhibitor may require a tRNA m^7^G pharmacodynamic assay, whereas an internal mRNA strategy may require site-specific measurement of the intended transcript. Clinical development should initially prioritize molecularly defined settings with convergent evidence, such as METTL1-high tumors with a demonstrated codon-biased program or treatment-induced writer dependence. These criteria can separate plausible therapeutic vulnerability from the much broader observation that m^7^G-associated proteins are dysregulated in cancer.

Experience with eIF4E illustrates both the promise and the difficulty of targeting an essential cap-recognition process. Therapeutic strategies have included antisense oligonucleotides, cap mimetics, kinase-pathway inhibition, and disruption of eIF4E-protein interactions, but selectivity for oncogenic translation remains a central requirement (Carroll and Borden, 2013; Karaki et al., 2015). Comparable development of METTL1-WDR4 inhibitors will require direct catalytic assays, structural confirmation, and biomarkers that distinguish tumor dependency from normal tRNA maintenance.

#### 5.3.1 Normal-tissue constraints and tumor-selective dependency

METTL1 inhibition may perturb tRNA maintenance and translation in normal proliferating tissues, hematopoietic and immune cells, and regenerating epithelia (PMID: 29983320; 39638793; 32391142; 41360736). Effects on stress tolerance and tissue recovery may become apparent during combination treatment even when baseline toxicity is limited (PMID: 38977661; 41360736). These are mechanistically plausible risks rather than established clinical toxicities of selective METTL1 inhibitors. Matched tumor and normal models should compare modification loss, translation changes, recovery after treatment, and immune function at equivalent exposure. A useful therapeutic window depends on tumor reliance on a defined substrate program; elevated writer expression alone is insufficient (PMID: 34352207; 34352206). Tumor-selective delivery or downstream transcript targeting may reduce systemic disruption, but selectivity must be demonstrated for each intervention.

#### 5.3.2 Compensation and resistance to pathway inhibition

Potential compensation includes increased abundance of alternative decoding tRNAs, altered codon demand, bypass through translation initiation, changes in reader availability, and metabolic or receptor-pathway rewiring (PMID: 34352207; 34352206; 37379838; 38280546; 37821451). These possibilities should be treated as hypotheses until demonstrated under sustained pathway inhibition. Persistence of the intended modification loss with recovery of the adaptive protein program would indicate downstream bypass; recovery of modification would instead suggest restored pathway activity or inadequate target engagement. Longitudinal RNA-class-specific mapping and translation measurements can distinguish these routes from loss of drug exposure (PMID: 38566310; 32514182; 29983320). Combination selection should follow the demonstrated escape mechanism while preserving normal-tissue recovery.

## 6 Conclusion and outlook

M^7^G has emerged as a distributed regulatory network that links RNA structure and fate to the adaptive demands of cancer. The most mature evidence supports METTL1-WDR4-dependent tRNA m^7^G46 as a determinant of tRNA stability and codon-biased translation. This mechanism enables tumors to increase selected proteins without requiring a uniform change in protein synthesis and provides a coherent explanation for diverse effects on cell-cycle progression, receptor signaling, metastasis, metabolism, DNA repair, stress tolerance, and immune-cell recruitment. RNMT-dependent cap methylation, rRNA-associated m^7^G, internal mRNA modification, circRNAs, and tRNA-derived fragments extend this system but occupy different levels of mechanistic certainty.

Several unresolved evidence questions now shape the field. First, expression of METTL1, WDR4, RNMT, or WBSCR22 cannot by itself identify the relevant RNA substrate or establish catalytic causality. Second, internal mRNA m^7^G requires especially rigorous validation because cap contamination, abundant tRNA and rRNA, antibody variability, and indirect transcript changes can all distort mapping. Third, the tumor-restrictive breast cancer phenotype demonstrates that increased tRNA m^7^G is not inevitably oncogenic. The biological direction depends on the modified tRNA repertoire, codon usage of available transcripts, reader context, and cellular state. Finally, WDR4 can regulate immune cells independently of m^7^G, showing that protein depletion and catalytic inhibition are not interchangeable experimental or therapeutic interventions. Treatment response is determined by the modified substrate, reader, and adaptive protein program; increased METTL1 expression or total m^7^G abundance alone does not predict resistance.

Progress will require integrating chemical mapping with functional genomics. Quantitative site-resolved measurements should be combined with ribosome profiling, tRNA abundance, codon-demand analysis, proteomics, and catalytic rescue. Single-cell and spatial approaches are needed to distinguish tumor-cell m^7^G programs from those operating in fibroblasts, macrophages, and other immune populations. Patient-derived organoids, immunocompetent models, and longitudinal samples collected before and after therapy can determine whether m^7^G changes initiate resistance or merely accompany it.

A common causal framework should distinguish regulator association, catalytic dependence, substrate validation, and downstream rescue. Candidate mechanisms should advance from regulator expression to catalytic dependence, site-resolved substrate validation, altered RNA fate, and phenotypic rescue, with each step tested in models that preserve cellular origin and an intact immune compartment. Dissociation between writer perturbation, modification occupancy, and protein output should be reported, because it can reveal non-catalytic functions or compensatory pathways. Such standards would reduce the tendency to treat prognostic signatures or global antibody signals as proof of an m^7^G-driven pathway and would make apparently conflicting tumor-promoting and tumor-restrictive observations easier to reconcile.

Therapeutic development should be guided by defined dependencies rather than pan-cancer writer overexpression. Selective inhibitors and degraders need structural validation, normal-tissue toxicology, and pharmacodynamic assays that report modification loss in the relevant RNA class. RNA-directed strategies may provide an alternative when a single modified transcript or non-coding RNA drives disease. Combination regimens are especially compelling because m^7^G programs repeatedly support DNA repair, kinase-inhibitor escape, metabolic adaptation, and immunosuppression. The central opportunity is to translate a compartment-specific RNA mechanism into a patient-specific treatment hypothesis while preserving the essential functions of m^7^G in normal RNA metabolism.
